# Injuries to High School Football Players in Long Island, New York: A Five-Year Study

**DOI:** 10.7759/cureus.115861

**Published:** 2026-09-06

**Authors:** Brian D Greenstein, Matthew A Zinner, Eric V Neufeld, William DeGouveia, Michael J Meade, Andrew Cornicello, Steven E Rokito

**Affiliations:** 1 Orthopedic Surgery, Donald and Barbara Zucker School of Medicine at Hofstra/Northwell, Hempstead, USA; 2 Orthopedic Surgery, Northwell Health, New Hyde Park, USA

**Keywords:** concussion, epidemiology, high school football, injury prevention, sports medicine

## Abstract

Background

American football has the highest injury rate of any U.S. high school sport, yet most studies examine a single injury type or position. We characterized injury patterns among high school football athletes in Long Island, New York, over five consecutive seasons, focusing on how injury patterns vary by level of play, setting, and position.

Methods

In this retrospective cohort study, certified athletic trainers and sports medicine physicians documented all in-season injuries among varsity and junior varsity (JV) athletes at 16 high schools from 2019 to 2023. Level of play, setting, mechanism, anatomic location, diagnosis, position, and time to return to play were recorded. Proportions were compared using two-sample Z-tests for independent proportions, with a two-sided p < 0.05 considered significant. Test statistics are reported as Z values alongside exact p-values.

Results

Among 3,861 athletes (2,093 varsity, 54.2%), 1,476 injuries were recorded. Varsity athletes accounted for a greater share of injuries than JV athletes (n = 1,104, 74.8% vs. n = 372, 25.2%; Z = 26.95, p < 0.001). Most injuries occurred in games despite far more practices (n = 732 of 1,308 injuries with setting recorded, 56.0%; Z = 6.10, p < 0.001). Direct contact was the leading mechanism (n = 796 of 1,344 injuries with the mechanism recorded, 59.2%). The lower extremity was most often injured (n = 672, 45.5%), followed by the upper extremity (n = 431, 29.2%) and head/neck (n = 187, 12.7%). Concussion was the most common specific diagnosis (n = 131, 8.9%). Profiles differed by position: offensive linemen had the highest proportion of concussions, and linebackers had the highest proportion of contact-related injuries. The proportion of athletes with a recorded concussion did not differ between varsity and JV athletes (Z = 0.53, p = 0.594).

Conclusions

A greater proportion of recorded injuries occurred among varsity athletes, in games, and through direct contact. Injury profiles differed by position, most notably a high proportion of concussions among offensive linemen. Because athlete exposure denominators were unavailable, these findings describe the distribution of recorded injuries rather than exposure-adjusted risk. These multiyear, position-specific data can inform targeted prevention and safety policy in high school football.

## Introduction

American football is the most popular sport among U.S. high school athletes, with more than one million participants in the 2022-2023 season [1]. While participation offers well-documented benefits, including improved cardiovascular fitness, coordination, and social development, it also carries a substantial risk of injury [2]. Among all high school sports, football has the highest reported injury rate [3,4].

Much of the recent attention to football safety has centered on concussion and its potential long-term neurologic effects, which has driven important rule and policy changes [4]. Far less characterized is the sport’s broader acute injury profile: which injuries occur, where on the body, and to whom. Most epidemiologic studies have examined a single position or injury type, failing to describe how injuries are distributed across positions, level of play, and setting of play.

Understanding this heterogeneity matters because the physical demands of football vary widely within a single roster. Linemen engage in repetitive line-of-scrimmage contact on nearly every play, whereas running backs (RBs), receivers, and defensive backs (DBs) accelerate, cut, and tackle in the open field, and these differing demands plausibly produce distinct injury profiles by position. Level and setting of play add a further dimension; the faster, more physical varsity level and game setting may carry different risks than participation at the junior varsity (JV) level or in practice.

To address these gaps, we performed a retrospective analysis of injury patterns among varsity and JV high school football athletes in Long Island, New York, over five consecutive seasons. The primary objective was to describe how recorded injuries were distributed by level of play, setting, and position. This objective was descriptive; because athlete exposure data were unavailable, the study was not designed to estimate injury rates or establish causal relationships.

## Materials and methods

We conducted a retrospective cohort study of all football athletes at 16 high schools in Long Island, New York, during the 2019-2023 seasons. Injuries were diagnosed at the time of occurrence by certified athletic trainers (ATCs), fellowship-trained orthopedic sports medicine physicians, and nonoperative sports medicine physicians present at practices and games and were documented as part of routine clinical care. Apart from concussion assessment, no standardized surveillance instrument or reporting platform was used across the participating schools. For each injury, we recorded the date, setting (game vs. practice), level of play (varsity vs. JV), mechanism, anatomic location, diagnosis, athlete position, and time to return to play. An injury was defined as any condition sustained during organized football participation that required evaluation by an ATC or physician and resulted in at least some restriction from participation. Concussions were identified using a standardized sideline assessment instrument (Sport Concussion Assessment Tool) administered by the school ATC, with diagnosis confirmed by the treating sports medicine physician in accordance with contemporaneous consensus guidelines.

Positions were classified as quarterback (QB), RB, wide receiver/tight end (WR/TE), offensive lineman (OL), defensive lineman (DL), DB, linebacker, and kicker/punter (K/P). Injury management and return-to-play decisions were made by each school’s ATC and treating physician. With the exception of the COVID-19-shortened 2020 season (eight weeks), each season comprised 10-12 weeks of regular play followed by two weeks of playoffs. Teams averaged five practices and one game per week. Injuries sustained in the preseason, offseason, or outside of football activity were excluded. Team rosters for each school and season were obtained from participating schools and used to determine the number of athletes at each level of play and average team size.

Proportions were compared using two-sample Z-tests for independent proportions with a pooled variance estimate; a two-sided p < 0.05 was considered statistically significant, and test statistics were reported as Z values alongside exact p-values. Continuous data are summarized as median (IQR) and mean. No cohort matching was performed. Missing data were handled by complete-case analysis without imputation, and denominators therefore vary by variable: level of play and anatomic location were recorded for all 1,476 injuries, athlete grade for 1,475, mechanism for 1,344, setting for 1,308 (both mechanism and setting for 1,287), position for 1,286, and return-to-play duration for 504. Among the 131 concussions, setting was recorded for 116. Nonspecific entries (minor injury, other injury, and unspecified pain) were excluded from diagnosis-specific analyses. To assess the potential influence of incomplete recording, we compared the proportion of missing values across injury types and levels of play and performed bounding analyses in which all unrecorded values were assigned to a single category.

This study was approved as an exempt protocol by the Northwell Health Human Research Protection Program (approval 24-1086), and the requirement for informed consent was waived. The investigation was conducted in accordance with the ethical principles for medical research involving human subjects set out in the World Medical Association Declaration of Helsinki (2013 revision).

## Results

Athlete and injury demographics

Over the five seasons, 3,861 athletes were included, of whom 2,093 (54.2%) played varsity, and 1,768 (45.8%) played JV (Table 1). Varsity teams were slightly larger than JV teams (mean, 32 vs. 27 athletes). A total of 1,476 injuries were recorded. Varsity athletes accounted for a significantly greater share of injuries (n = 1,104, 74.8%) than their JV counterparts (n = 372, 25.2%; Z = 26.95, p < 0.001).

**Table 1:** Demographics of athletes and injuries observed. Data are presented as n (%) unless otherwise indicated; average team size is presented as the mean. Comparisons between varsity and JV were performed using Z-tests for proportions. * Statistically significant at p < 0.05 (Z = 26.95, p < 0.001). JV, junior varsity

Level of play	Number of athletes, n (%)	Number of injuries, n (%)	Average team size (athletes)
Varsity	2,093 (54.2)	1,104 (74.8)*	32
JV	1,768 (45.8)	372 (25.2)*	27
Total	3,861 (100)	1,476 (100)	29.5

Consistent with the composition of each level, injuries among seniors and juniors occurred predominantly at the varsity level, whereas injuries among sophomores and freshmen occurred predominantly at the JV level (Figure 1).

**Figure 1:**
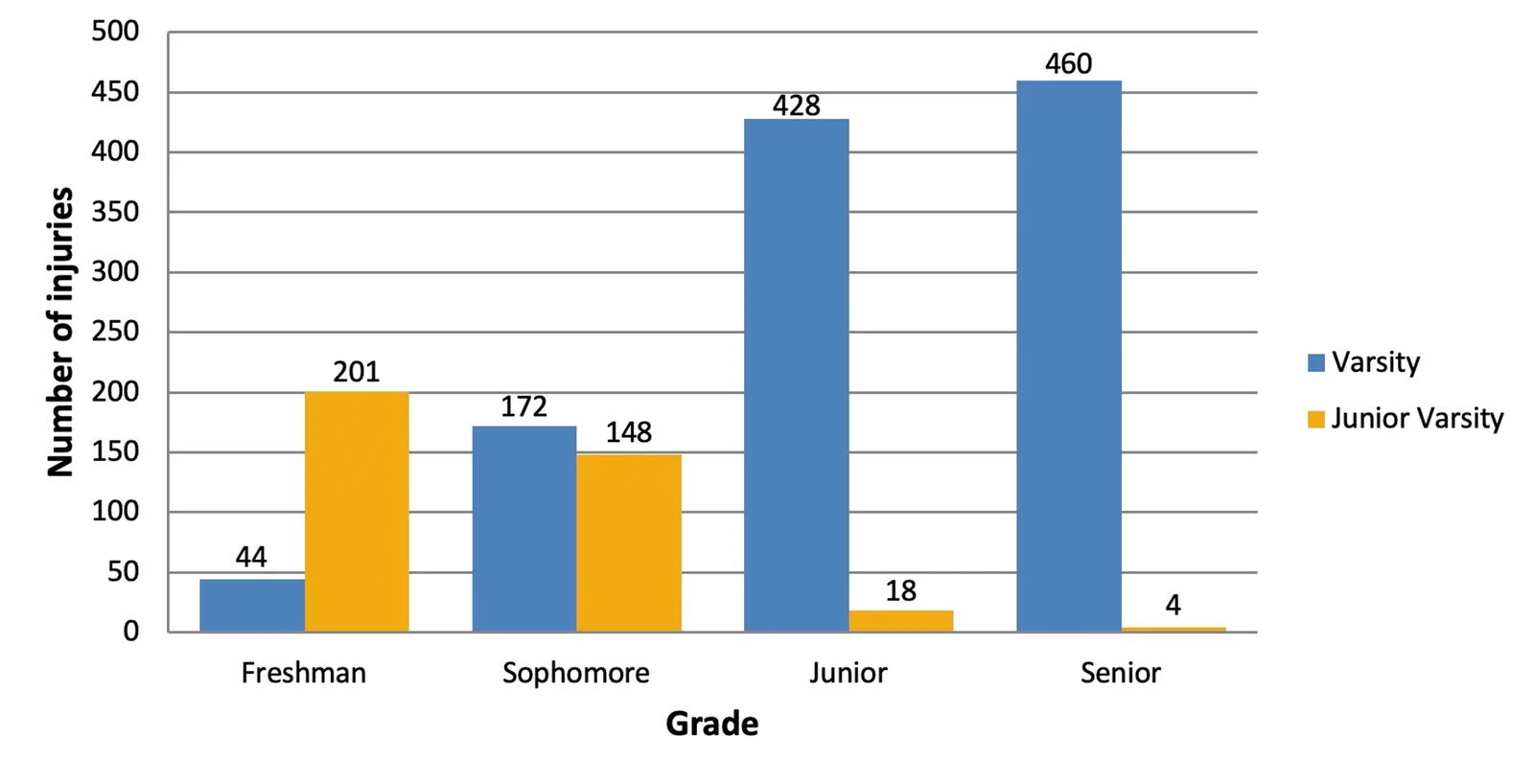
Distribution of injury count stratified by grade and level of play. Data are presented as raw counts. One JV injury had no grade recorded and was omitted from this distribution. JV, junior varsity

Injury setting

A greater proportion of recorded injuries occurred in games than in practices; despite roughly five times as many practices as games, most injuries occurred during games (n = 732, 56.0%; Z = 6.10, p < 0.001). This pattern was more pronounced at the varsity level, where a greater proportion of injuries occurred in games (n = 570, 58.6%) than at the JV level (n = 162, 48.2%; Z = 3.32, p < 0.001).

Mechanism of injury

Direct contact was the most common mechanism of injury (n = 796, 59.2%), accounting for more than twice as many injuries as noncontact mechanisms (n = 366, 27.2%; Z = 16.74, p < 0.001). The remaining 182 injuries (13.5%) had an insidious onset. The mechanism of injury varied by setting of play (Figure 2). Contact caused a greater proportion of game injuries (n = 508, 70.8%) than practice injuries (n = 276, 48.5%; Z = 8.12, p < 0.001). Conversely, fewer game injuries were noncontact (n = 167, 23.3%) than practice injuries (n = 183, 32.2%; Z = -3.56, p < 0.001). Among contact injuries, player-to-player contact was far more common than contact with an object such as the ball or ground.

**Figure 2:**
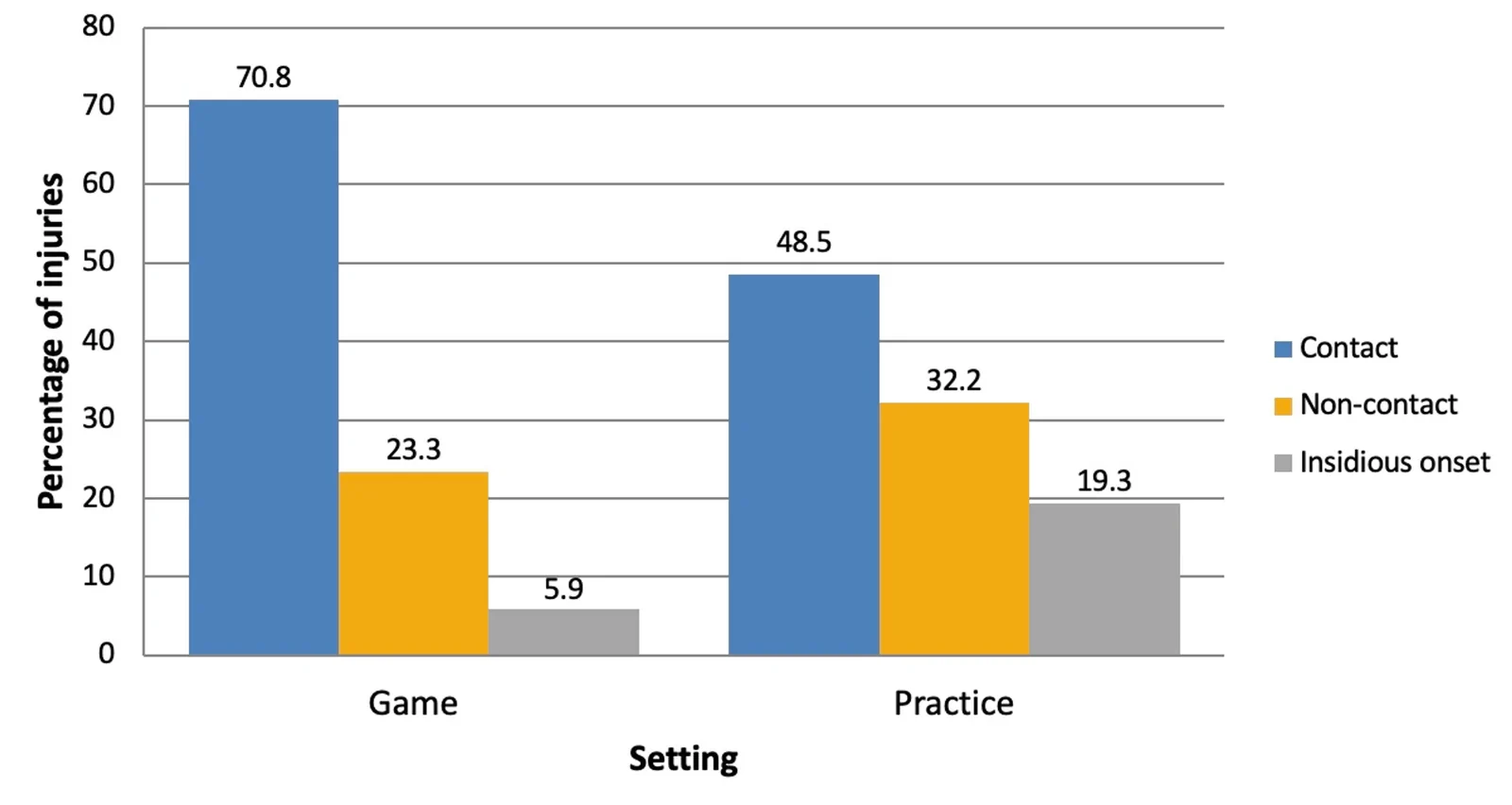
Distribution of injury mechanism in games and practices. The mechanism of injury was categorized as contact, noncontact, or insidious onset. Data are presented as the percentage of injuries within each setting of play (game, n = 718; practice, n = 569). Comparisons were performed using Z-tests for proportions; significance was set at p < 0.05.

Anatomic distribution of injuries

The lower and upper extremities accounted for the majority of injuries (n = 672, 45.5% and n = 431, 29.2%, respectively), followed by the head/neck (n = 187, 12.7%) (Table 2). The overall distribution was similar across levels, with the exception of head/neck injuries, which comprised a greater proportion of injuries at the JV level (n = 69, 18.5%) than at the varsity level (n = 118, 10.7%; Z = -3.94, p < 0.001), and hip/groin injuries, which comprised a greater proportion at the varsity level (n = 67, 6.1%) than at the JV level (n = 8, 2.2%; Z = 2.98, p = 0.003). The most frequently injured body parts were the knee (n = 222, 15.0%), ankle (n = 201, 13.6%), hand/fingers (n = 169, 11.4%), head (n = 151, 10.2%), and shoulder/clavicle (n = 139, 9.4%). Across all settings, levels, and positions, the two most common specific diagnoses were concussion and tear of the lateral ankle ligament complex.

**Table 2:** Anatomic distribution of injuries by level of play. Data are presented as n (%) within each level of play; percentages in the “All athletes” column are of all 1,476 injuries. Totals: varsity, n = 1,104; JV, n = 372; all athletes, n = 1,476. Comparisons between varsity and JV were performed using Z-tests for proportions, with significance set at p < 0.05. JV, junior varsity

Anatomic region	Varsity, n (%)	JV, n (%)	All athletes, n (%)	Z	p-value
Lower extremity	497 (45.0)	175 (47.0)	672 (45.5)	-0.68	0.498
Upper extremity	335 (30.3)	96 (25.8)	431 (29.2)	1.66	0.096
Head/neck	118 (10.7)	69 (18.5)	187 (12.7)	-3.94	<0.001
Hip/groin	67 (6.1)	8 (2.2)	75 (5.1)	2.98	0.003
Back	57 (5.2)	13 (3.5)	70 (4.7)	1.31	0.19
Chest/ribs	17 (1.5)	8 (2.2)	25 (1.7)	-0.79	0.43
Other	8 (0.7)	3 (0.8)	11 (0.7)	-0.16	0.874
Abdomen	5 (0.5)	0 (0)	5 (0.4)	1.3	0.194

The most common lower extremity injury was a lateral ankle ligament complex tear, documented 110 times, more than four times as often as any other lower extremity injury (Table 3). Anterior cruciate ligament tears, high ankle sprains, and medial collateral ligament (MCL) tears followed, occurring 26, 24, and 23 times, respectively.

**Table 3:** Five most common lower extremity diagnoses at each anatomic location. Each cell gives the diagnosis followed by the number of injuries. Diagnoses are ranked in descending order of frequency. Nonspecific entries (minor injury, other injury, and unspecified pain) were excluded. Total injuries by location: knee, n = 222; ankle, n = 201; lower leg/Achilles, n = 87; thigh, n = 86. ACL, anterior cruciate ligament; MCL, medial collateral ligament

Rank	Knee	Ankle	Lower leg/Achilles	Thigh
1	ACL tear, 26	Lateral ligament complex tear, 110	Shin splints, 15	Hamstring tear, 21
2	MCL tear, 23	High ankle sprain, 24	Fibula fracture, 13	Quadriceps contusion, 11
3	Patellar tendinitis, 18	Medial (deltoid) ligament tear, 7	Achilles tendonitis, 10	Quadriceps strain, 8
4	Patella subluxation/dislocation, 15	Lateral malleolus fracture, 2	Contusion, 6	Hamstring spasm, 5
5	Lateral meniscus tear, 12	Dislocation, 2	Tibia fracture, 4	Hamstring tendinitis, 3

The most common upper extremity injury was an acromioclavicular (AC) joint sprain, recorded 42 times (Table 4). Hand injuries (proximal and distal interphalangeal joint sprains, contusions, and metacarpophalangeal joint sprains) and radius fractures were the next most frequent.

**Table 4:** Five most common upper extremity diagnoses at each anatomic location. Each cell gives the diagnosis followed by the number of injuries. Diagnoses are ranked in descending order of frequency. Nonspecific entries (minor injury, other injury, and unspecified pain) were excluded. Wrist and forearm are combined because only two specific diagnoses were recorded for the forearm. Total injuries by location: hand/fingers, n = 169; shoulder/clavicle, n = 139; wrist/forearm, n = 85; elbow, n = 31. AC, acromioclavicular; MCP, metacarpophalangeal; UCL, ulnar collateral ligament

Rank	Hand/fingers	Shoulder/clavicle	Wrist/forearm	Elbow
1	Phalangeal sprain, 29	AC joint sprain, 42	Radius fracture, 18	UCL tear, 3
2	Contusion, 19	Clavicle fracture, 13	Scaphoid fracture, 5	Tendonitis, 2
3	MCP sprain, 17	Anterior dislocation, 13	Flexor tenosynovitis, 3	Dislocation, 1
4	Metacarpal fracture, 12	Anterior subluxation, 13	Ulna fracture, 2	Ulnar nerve subluxation, 1
5	Phalangeal fracture, 11	Rotator cuff strain, 10	Flexor tendon tear, 2	Lateral epicondylitis, 1

Injuries by position

Offensive players accounted for the majority of injuries (n = 779, 60.6%), followed by defensive players (n = 492, 38.3%) and K/P (n = 15, 1.2%). Among offensive players, WR/TE sustained the most injuries (n = 259, 33.2%), followed by OL (n = 233, 29.9%), RB (n = 202, 25.9%), and QB (n = 85, 10.9%). Among defensive players, DL sustained the most injuries (n = 196, 39.8%), followed by LB (n = 193, 39.2%) and DB (n = 103, 20.9%).

Lower extremity injuries predominated at every position (Figure 3). LB had the lowest proportion of lower extremity injuries (n = 81, 42.0%), and K/P had the highest (n = 9, 60.0%). RB were more than twice as likely to have a lower extremity injury than an upper extremity injury (n = 105, 52.0% vs. n = 39, 19.3%; Z = 6.86, p < 0.001). Apart from K/P, the upper extremity was the second most commonly injured region for all positions. OL had the highest proportion of head/neck injuries, twice that of DL (n = 43, 18.5% vs. n = 18, 9.2%; Z = 2.74, p = 0.006), with RB (n = 31, 15.3%) and LB (n = 30, 15.5%) also sustaining relatively high proportions of head/neck injuries. K/P had the highest proportion of hip/groin injuries (n = 4, 26.7%), and LB had the highest proportion of chest/rib injuries (n = 9, 4.7%).

**Figure 3:**
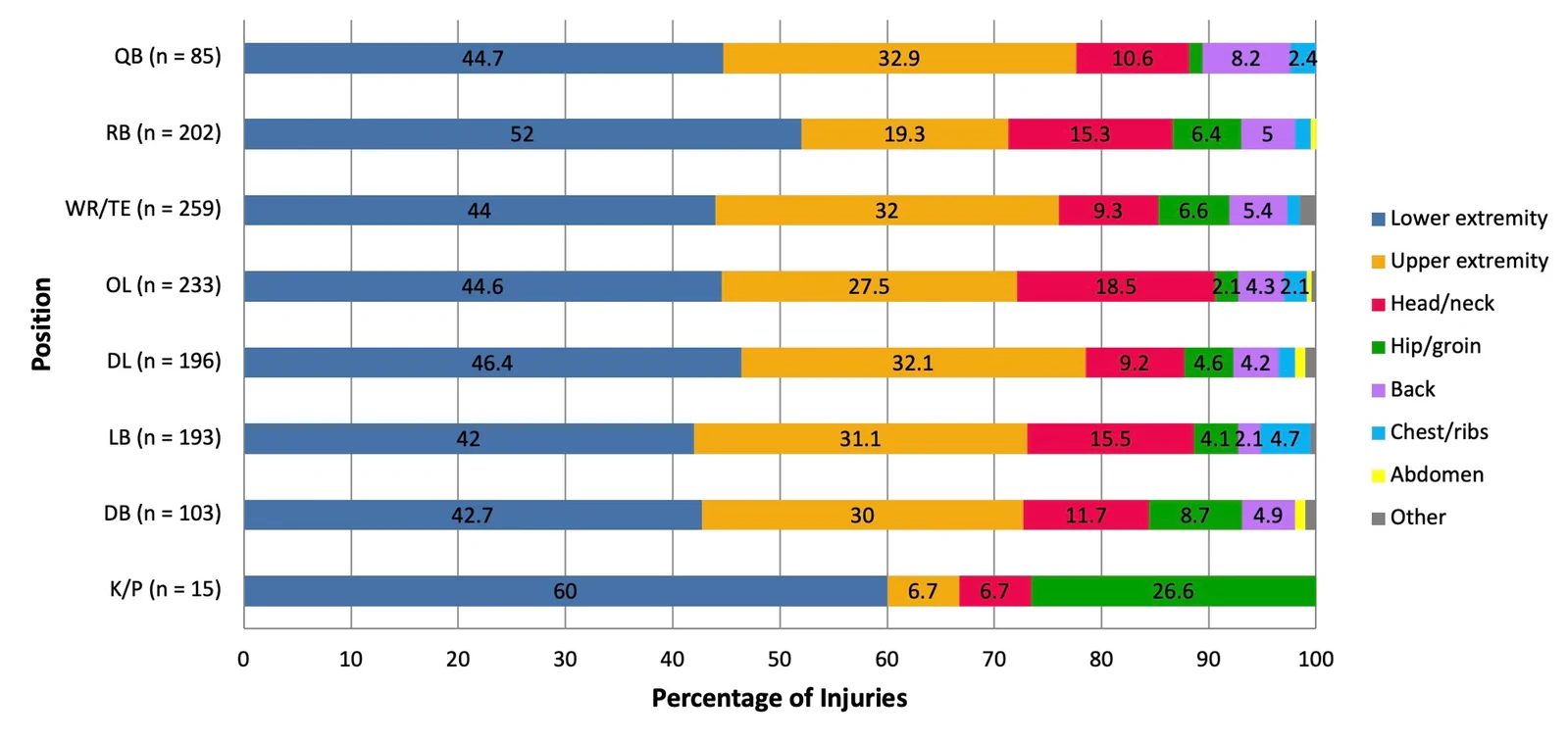
Anatomic distribution of injuries by position. Data are presented as the percentage of injuries at each position, rounded to whole numbers. The number of injuries at each position is shown on the vertical axis. Anatomic areas comprising greater than 2% of injuries at a position are labeled. Comparisons were performed using Z-tests for proportions; significance was set at p < 0.05. DB, defensive back; DL, defensive lineman; K/P, kicker/punter; LB, linebacker; OL, offensive lineman; QB, quarterback; RB, running back; WR/TE, wide receiver/tight end

Concussion was the most common injury for LB, OL, QB, and RB (Table 5). For LB and OL, concussions were twice as common as any other injury. Lateral ankle ligament complex tears were the most common injury for DB, DL, and WR/TE. QB was the only position for which an injury other than concussion or lateral ankle ligament complex tear, namely, AC joint sprain, ranked among the two most common injuries. AC joint sprain was the third most common injury for DB and the fourth most common for DL and LB, while a ligamentous injury of the knee was the third most common for DL, OL, and LB. For WR/TE, radius, fibula, and clavicle fractures were tied as the third most common injuries.

**Table 5:** Five most common injuries for each position. Each cell gives the diagnosis followed by the number of injuries. Diagnoses are ranked in descending order of frequency. Nonspecific entries (minor injury, other injury, and unspecified pain) were excluded. Total injuries by position: DB, n = 103; defensive lineman, n = 196; linebacker, n = 193; OL, n = 233; QB, n = 85; running back, n = 202; WR/tight end, n = 259; K/P, n = 15. Position was recorded for 1,286 of 1,476 injuries. AC, acromioclavicular; ACL, anterior cruciate ligament; DB, defensive back; DL, defensive lineman; K/P, kicker/punter; LB, linebacker; MCL, medial collateral ligament; OL, offensive lineman; QB, quarterback; RB, running back; TE, tight end; WR, wide receiver

Position	Rank 1	Rank 2	Rank 3	Rank 4	Rank 5
DB	Lateral ankle ligament complex tear, 11	Concussion, 7	AC joint sprain, 4	Shin splints, 4	Hip flexor tear, 3
DL	Lateral ankle ligament complex tear, 19	Concussion, 8	ACL tear, 6	AC joint sprain, 6	MCL tear, 4
LB	Concussion, 20	Lateral ankle ligament complex tear, 10	MCL tear, 8	AC joint sprain, 6	Anterior shoulder subluxation, 5
OL	Concussion, 30	Lateral ankle ligament complex tear, 12	MCL tear, 6	Patella subluxation, 6	Patellar tendinitis, 6
QB	Concussion, 9	AC joint sprain, 5	Lateral ankle ligament complex tear, 4	Hamstring tear, 3	Anterior shoulder dislocation, 2
RB	Concussion, 22	Lateral ankle ligament complex tear, 21	High ankle sprain, 6	Hamstring tear, 5	Shin splints, 5
WR/TE	Lateral ankle ligament complex tear, 20	Concussion, 17	Radius fracture, 6	Fibula fracture, 6	Clavicle fracture, 6
K/P	Lateral ankle ligament complex tear, 2	Concussion, 1	Adductor (groin) tear, 1	Hip flexor tear, 1	Quadriceps tear, 1

Every position except K/P sustained a greater proportion of injuries through direct contact than through noncontact or insidious mechanisms (Figure 4). Contact accounted for more than 60% of injuries among LB (n = 131, 67.9%), QB (n = 56, 65.9%), OL (n = 147, 63.1%), and DL (n = 123, 62.8%). K/P and WR/TE had the largest proportions of noncontact injuries (n = 7, 46.7% and n = 86, 33.2%, respectively), although the number of K/P injuries was small (n = 15).

**Figure 4:**
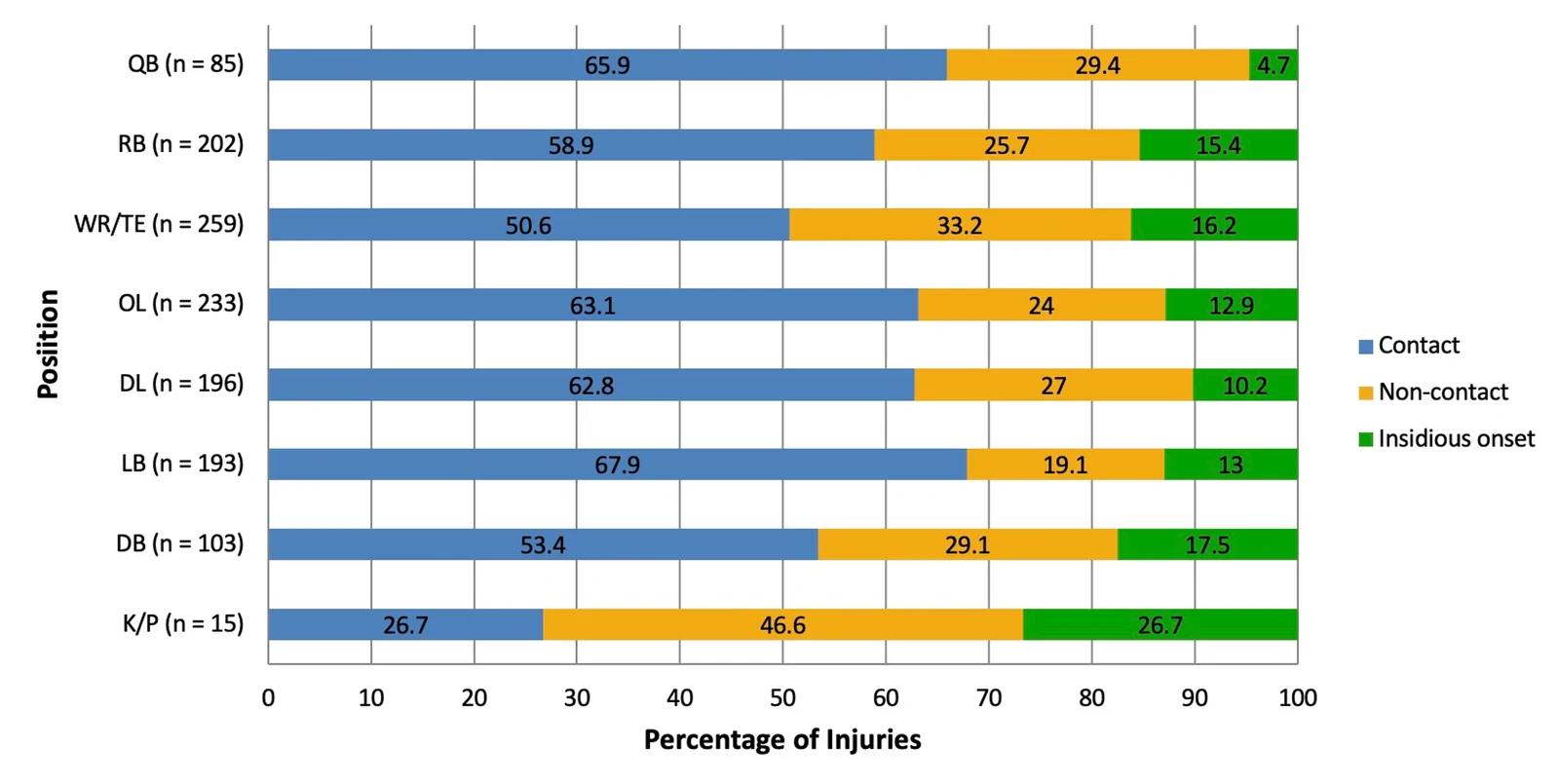
Mechanism of injury by position. Injuries were categorized as contact, noncontact, or insidious onset. Data are presented as the percentage of injuries at each position, rounded to whole numbers. The number of injuries at each position is shown on the vertical axis. DB, defensive back; DL, defensive lineman; K/P, kicker/punter; LB, linebacker; OL, offensive lineman; QB, quarterback; RB, running back; TE, tight end; WR, wide receiver; WR/TE, wide receiver/tight end

Concussions

A total of 131 concussions were recorded, representing 8.9% of all injuries and making concussion the most common specific diagnosis. Concussions occurred more often in games (n = 79, 68.1%) than in practices (n = 37, 31.9%; Z = 5.51, p < 0.001). The proportion of athletes with a recorded concussion did not differ significantly between varsity and JV athletes (n = 74, 3.5% vs. n = 57, 3.2%; Z = 0.53, p = 0.594). Offensive linemen sustained the most concussions (n = 30), followed by RB (n = 22), LB (n = 20), and WR/TE (n = 17). Among the 41 concussions with a recorded return-to-play duration, the median time lost was 14 days (IQR, 12-20; mean, 18 days).

Sensitivity analysis

Documentation completeness did not differ between concussions and all other injuries (setting missing for 15 of 131 concussions (11.5%) vs. 153 of 1,345 other injuries (11.4%); Z = 0.03, p = 0.979; position missing for 17 (13.0%) vs. 173 (12.9%); Z = 0.04, p = 0.970), nor between fractures, ligament tears, and dislocations (n = 445) and other diagnoses (n = 1,031; all (|Z| < 0.6), all (p > 0.57)). Missingness was modestly higher among varsity than JV injuries for mechanism (109 of 1,104 (9.9%) vs. 23 of 372 (6.2%); Z = 2.16, p = 0.031) and position (152 (13.8%) vs. 38 (10.2%); Z = 1.77, p = 0.077). In bounding analyses assigning all unrecorded values to a single category, the proportion of injuries occurring in games ranged from 49.6% to 61.0%, and the proportion attributed to direct contact ranged from 53.9% to 62.9%. The greater proportion of contact injuries in games than in practices persisted under the most adverse assumption (Z = 7.44, p < 0.001), whereas the difference in game-related injury proportions between varsity and JV athletes did not (Z = -0.53, p = 0.594).

## Discussion

Football is among the most popular and most hazardous sports played by U.S. adolescents. In this five-season analysis of high school football in Long Island, New York, the distribution of recorded injuries varied meaningfully by level of play, setting, and position, reinforcing that injury patterns are not uniform across the sport.

The most frequently injured body parts (knee, ankle, hand, head, and shoulder) are consistent with prior high school football epidemiology [5-7], and the predominance of knee and ankle injuries mirrors sport-wide high school surveillance, in which these are the most commonly injured sites [8,9]. Beyond confirming these patterns, our data show that susceptibility to specific injuries differs by context. Varsity athletes accounted for a disproportionate share of injuries relative to their representation in the cohort, and injuries were more common in games than in practices. Because varsity play and game settings are generally more intense than JV play and practices, these findings support an association between intensity of play and injury frequency.

Position-specific profiles were similarly distinct. Consistent with prior work, QBs had the highest proportion of shoulder injuries of any position (n = 11, 12.9%), and the shoulder was among their most frequently injured structures [10,11]. Earlier studies have identified running plays as a leading cause of injury, particularly lower-body injury among RB and LB [5,12]. Although we did not record play type, our finding that RB had the highest proportion of lower extremity injuries aside from K/P is consistent with this. WR/TE had the highest proportion of noncontact injuries among skill positions (n = 86, 33.2%), exceeded only by K/P (n = 7, 46.7%). LB, typically responsible for the most tackles and thus exposed to high levels of contact [13], had the highest proportion of contact-related injuries (n = 131, 67.9%). As expected, K/P sustained the fewest injuries. Their injuries were concentrated in the lower body, consistent with a prior study of National Football League kickers [14].

The most notable positional finding was the concussion burden among offensive linemen, who accounted for more recorded concussions than any other position and for whom concussion was more than twice as frequent as any other diagnosis. Linemen attract less concussion attention than skill positions, yet in-helmet sensor studies consistently show that linemen incur the highest frequency of head impacts in high school and collegiate football, reflecting the repetitive line-of-scrimmage contact that occurs on virtually every play [15-17]. Our clinically diagnosed data are consistent with this exposure signal, although without position-specific denominators we cannot determine whether linemen sustain concussions at a higher rate than athletes at other positions. This pattern diverges from per-athlete exposure analyses in professional football, where skill positions such as DBs and TEs show the highest concussion incidence [18], and from youth data reporting no position-based difference [19]. Together, these findings indicate that position-specific concussion risk remains unsettled and that linemen warrant closer surveillance. Concussion was the most common specific diagnosis overall, in agreement with previous high school surveillance [4,20-22]. We found no difference in the proportion of athletes with a recorded concussion between varsity and JV athletes; a prior report described longer symptom duration among JV athletes [23], which our data were not designed to assess.

This study has several limitations. Injuries were likely underreported, as not all injured athletes presented to the medical staff. Documentation was not standardized across the 16 participating schools, which may have contributed to this incomplete recording. The cohort was drawn from a single geographic area (Long Island, New York), which may limit generalizability; multiregion studies are needed to reduce sampling bias. Return-to-play duration was documented for only 34.1% of injuries, and we therefore did not report time-loss estimates for most injuries. Setting, mechanism, and position were likewise incompletely recorded, and all proportions use complete-case denominators. Sensitivity analyses showed no evidence of preferential documentation by injury severity. The excess of contact injuries in games held under the most unfavorable assumptions about the missing values; the varsity-versus-JV difference in game-related injuries did not, and that comparison should be interpreted cautiously. Finally, we lacked positional data for uninjured athletes and athlete exposure denominators, precluding calculation of position-specific injury rates and injury rates per athlete exposure.

## Conclusions

This five-year study characterizes recorded injuries among high school football players in Long Island, New York. A greater proportion of injuries occurred among varsity than JV athletes, in games than in practices, and through direct contact than through other mechanisms. The knee, ankle, hand, head, and shoulder were the most commonly injured body parts, and concussion was the most common specific diagnosis, most strikingly among offensive linemen, a group that has received less attention than skill positions in the concussion literature. Injury patterns were not uniform: each position and level of play showed a distinct profile of recorded injuries. Because athlete exposure data were unavailable, these findings describe patterns and proportions of recorded injuries rather than exposure-adjusted incidence or relative risk. As initiatives such as Heads Up Football and targeting rules illustrate, understanding these patterns and translating them into structural change can better protect athletes. These data can help coaches, medical staff, families, and players anticipate, recognize, and manage football-related injuries in the high school population.
